# Delayed diagnosis of type I jejunal atresia in an infant with intractable vomiting: a case report

**DOI:** 10.1093/jscr/rjaf429

**Published:** 2025-06-18

**Authors:** Hadjar Nassiri, Jaouad Bouljrouf, Monim Ochan, Mounir Kisra

**Affiliations:** Faculté de Medecine et de Pharmacie de Rabat, Université Mohammed V de Rabat. Av. Hafiane Cherkaoui, Rabat 10000, Morocco; Pediatric Surgery, Ibn Sina University Hospital Center, BP 6527, Rue Lamfadel Cherkaoui Rabat Institut, Rabat 1005, Morocco; Laboratory of Life and Health Sciences, Faculty of Medicine and Pharmacy of Tangier, Abdelmalek Essaadi University, La Nouvelle Ville Ibn Batouta, Tangier-Tetouan 90000, Morocco; Faculté de Medecine et de Pharmacie de Rabat, Université Mohammed V de Rabat. Av. Hafiane Cherkaoui, Rabat 10000, Morocco; Pediatric Surgery, Ibn Sina University Hospital Center, BP 6527, Rue Lamfadel Cherkaoui Rabat Institut, Rabat 1005, Morocco; Faculté de Medecine et de Pharmacie de Rabat, Université Mohammed V de Rabat. Av. Hafiane Cherkaoui, Rabat 10000, Morocco; Pediatric Surgery, Ibn Sina University Hospital Center, BP 6527, Rue Lamfadel Cherkaoui Rabat Institut, Rabat 1005, Morocco; Faculté de Medecine et de Pharmacie de Rabat, Université Mohammed V de Rabat. Av. Hafiane Cherkaoui, Rabat 10000, Morocco; Pediatric Surgery, Ibn Sina University Hospital Center, BP 6527, Rue Lamfadel Cherkaoui Rabat Institut, Rabat 1005, Morocco

**Keywords:** chronic vomiting, infant, jejunal atresia, intestinal obstruction, diagnosis delay

## Abstract

We report the case of a 14-month-old infant with chronic vomiting and recurrent subocclusive episodes since 40 days of life. Initially misdiagnosed as cow’s milk protein allergy and gastritis, the infant underwent repeated admissions for dehydration and failure to thrive. Radiologic studies demonstrated dilated proximal small bowel loops but no obvious cause for obstruction. Exploratory laparotomy revealed a type I jejunal atresia due to an incomplete mucosal diaphragm and two congenital fibrous bands contributing to intermittent obstruction. Resection of the segment and division of the bands with end-to-end anastomosis resulted in full clinical recovery and catch-up growth. This case highlights the importance of considering congenital structural anomalies, such as partial atresias and congenital bands, in the differential diagnosis of persistent gastrointestinal symptoms in infants, especially when these symptoms are unresponsive to medical treatment and radiologic studies suspicious of partial obstruction.

## Introduction

In infants, subocclusive episodes and chronic vomiting in infancy, can be so challenging to diagnose mostly because their nonspecific symptoms and due to a large differential diagnoses. We commonly consider causes such us cow’s milk protein allergy (CMPA), gastroesophageal reflux disease (GERD), viral gastroenteritis, or pyloric stenosis [1].

Usually, a trial of dietary changes or medical treatment results in resolution of symptoms. Still, rare structural abnormalities like congenital intestinal atresias have to be taken into account even beyond the newborn period when symptoms persist or worsen.

Congenital intestinal malformations, as the type I jejunal atresia, are usually diagnosed during the neonatal period, but partial or intermittent forms may escape early diagnosis and delay the treatment [2].

Jejunal or jejunoileal atresias differ from those located proximally in their aetiology, associated abnormalities, treatment and even prognosis [3]. They are more common than duodenal atresias and are caused by an ischaemic event during pregnancy, which can be caused by intussusception, perforation, volvulus, hernia or thromboembolism. The estimated incidence is 1–3 per 10 000 births [4]. There is no gender predisposition and it is uncommon for it to be accompanied by other malformations. [3].

This paper highlights a rare case of type I jejunal atresia with incomplete obstruction and congenital bands, only diagnosed after a prolonged symptomatic course and incorrect diagnoses and long ongoing treatment. This case demonstrates the need for a high index of suspicion and thorough investigation in infants with gastrointestinal symptoms.

## Case report

A 14-month-old infant was referred to our department for evaluation. He presents intractable vomiting and recurrent subocclusive syndromes that had been ongoing since the age of 40 days.

This child had been admitted to the paediatrics department several times for dehydration and non-specific digestive disorders. Normal meconium passage was reported at birth and no episode of full cessation of the stools was described.

The initial diagnostic work-up evocated cow’s milk protein allergy, then gastritis, but no clinical improvement occurred after diet changes or medical treatment.

**Figure 1:**
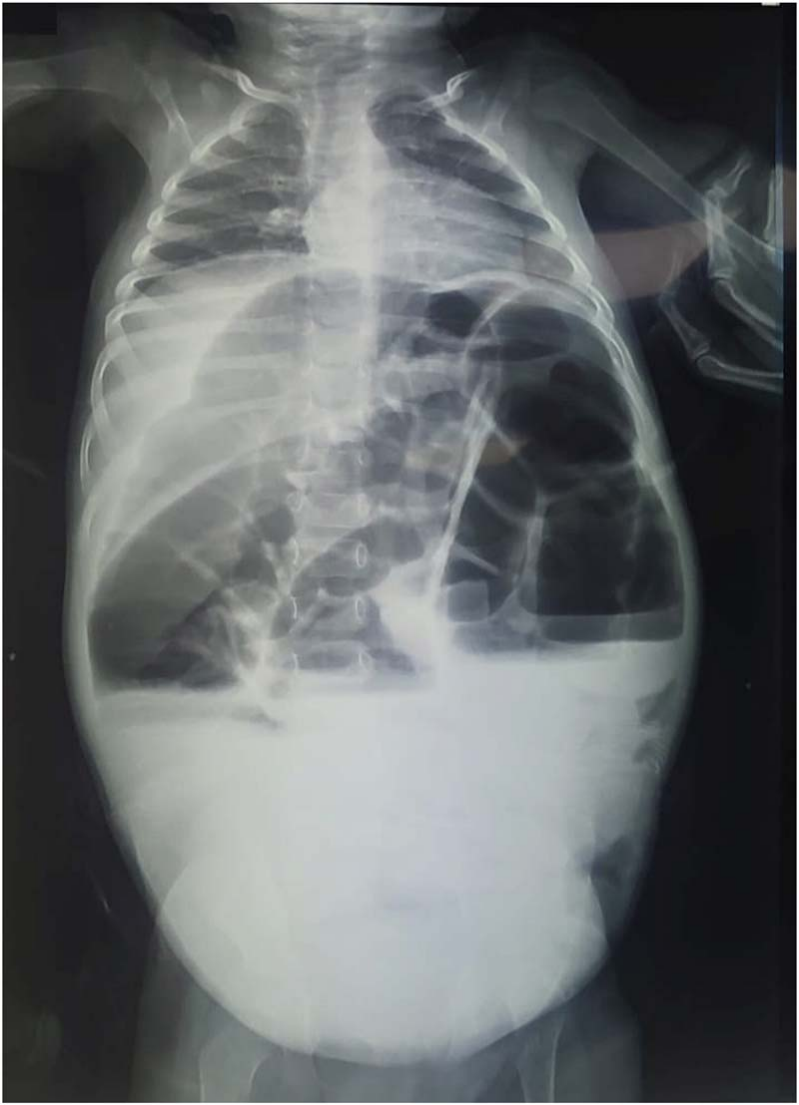
X-ray image showing intestinal-type hydroaeric levels and gastric stasis.

**Figure 2:**
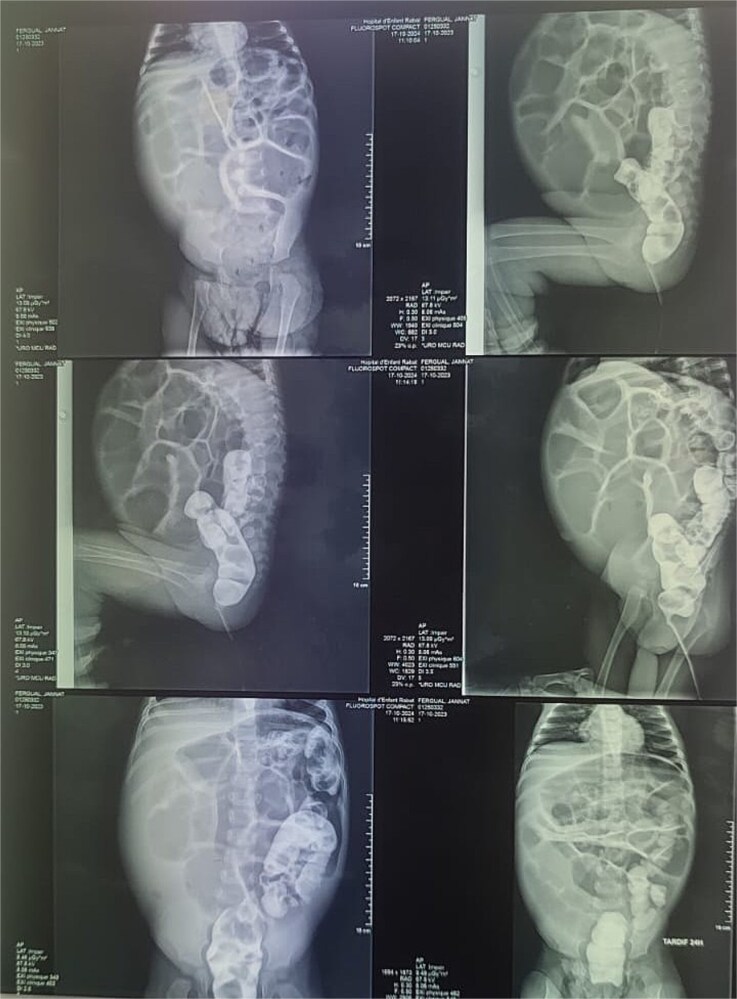
Barium index suggesting an upper intestinal obstruction.

**Figure 3:**
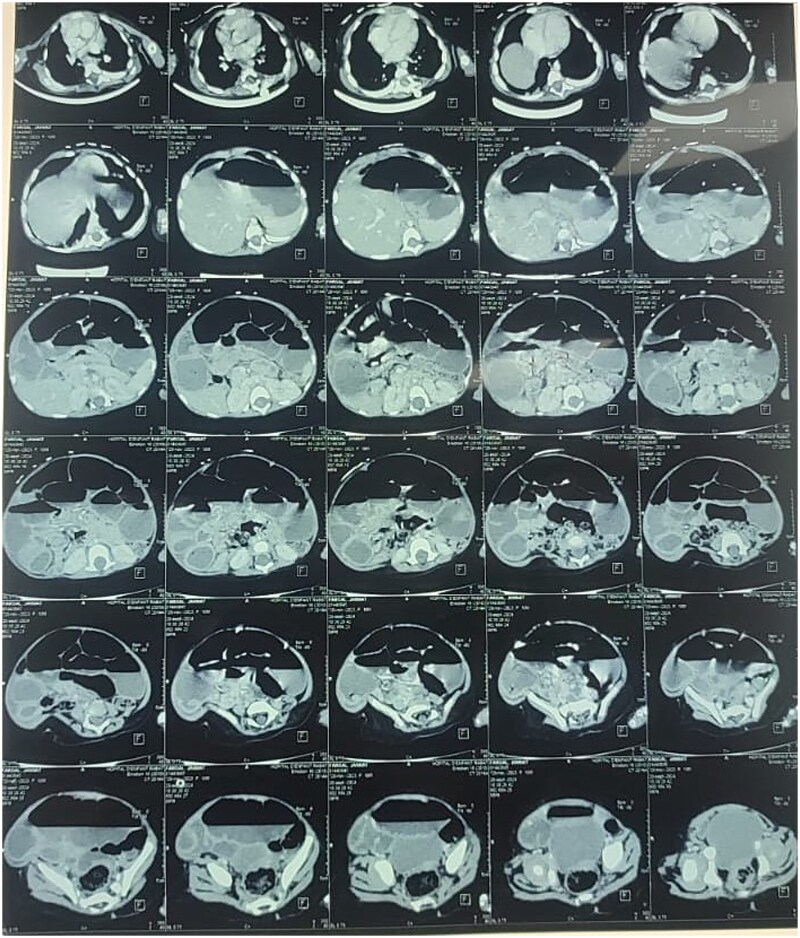
Enteroscan image showing an intestinal distension downstream of an obstacle.

Reviewing the medical record of the patient, she underwent variable blood tests that were negative mostly except for ionogram disturbances due to malnutrition and dehydration. An abdominal X-ray was performed at each episode of hospitalization, that revealed intestinal-type hydroaeric levels and gastric stasis (Fig. 1) then a digestive fibroscopy with multiple biopsis as well as eosogastroduodenal transit were run out suggesting gastritis. A barium index (Fig. 2) was also performed on his last hospitalization suggesting a small bowel obstruction which also appeared on the Enteroscan (Fig. 3).

On admission to the surgical department, the patient had significant growth retardation and dehydration, a distended abdomen with cessation of materials and gas, and bilious vomiting.

**Figure 4:**
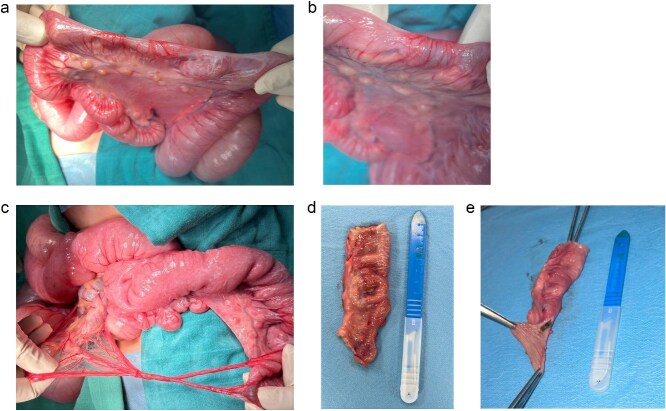
Surgery images. (a) Surgery imaging showing the type one jejunal atresia in our patient. (b) Surgery imaging showing the type one jejunal atresia in our patient (close up picture). (c) Congenital bands. (d and e) Resected segment carrying the diaphragm.

Due to inconclusive imaging and persistent clinical symptoms, an exploratory laparotomy was decided. The intraoperative findings were multiple type I jejunal atresia due to incomplete mucosal diaphragms, located exactly at the 6th jejunal loop and across which intestinal contents could pass only as a thread (Fig. 4a and b). Two congenital fibrous bands at the jejunum level were also observed, further probably contributing to intermittent obstruction (Fig. 4c).

A resection of the affected jejunal segment was done, including diaphragm and congenital bands. An end-to-end anastomosis was created (Fig. 4d and e).

The postoperative course was uneventful. Bowel function returned promptly. The infant was able to tolerate oral feeds. At follow-ups, intake of nutrition was improved and catch-up growth was observed.

## Discussion

Intestinal obstruction is common in the neonatal period and is typically classified as either high or low obstruction based on the radiographic bowel gas pattern [5].

Jejunal atresia, a congenital anomaly resulting from intrauterine mesenteric vascular accidents or developmental malformations, accounts for 20%–30% of all intestinal atresias, type I jejunal atresia for nearly 50% of jejunal atresias [2, 6]. It is usually diagnosed in the first days of life as a consequence of bilious vomiting and absence of meconium passage. Type I atresia can, however, present more insidiously, in particular when the obstruction diaphragm is incomplete and allows for limited passage of the intestinal content. The disease can, therefore, mimic GERD, CMPA, and infectious enteritis, as in our patient.

In our case, the early work up suggested CMPA and later gastritis. This misdirection led to delay in definitive management and continued chronic malnutrition and growth retardation. Similar diagnostic delays have been described in partial atresias elsewhere, which underpins the difficulty in identifying structural causes in the presence of functional symptoms [7, 8].

Radiologic examinations such as abdominal X-rays and contrast studies may show signs of incomplete obstruction, such as proximal bowel dilatation or delay, but may not detect mucosal diaphragms or extrinsic compressions in case of intermittent or partial obstruction [9]. Cross-sectional imaging, such as computed tomography or magnetic resonance enterography, can add value, yet intraluminal causes may not be identified without high clinical suspicion.

Congenital bands, fibrous remnants of embryologic structures such as the vitelline duct or mesenteric attachments, are another rare cause of small bowel obstruction in children [10]. These bands may be associated with other anomalies and are often recognized only at surgery. In our patient, two such bands contributed to the obstruction caused by the jejunal diaphragm and led to a complex, intermittently symptomatic obstruction. Surgical exploration is still both diagnostic and therapeutic when noninvasive modalities cannot identify the cause of obstruction. Recognition of the following intraoperative findings in our patient—a type I jejunal atresia with a fenestrated diaphragm and two congenital bands—demonstrates the need to consider structural causes early in the diagnostic algorithm for chronic vomiting in infants. The postoperative course is usually uneventful with appropriate resection and anastomosis as was the case for this patient.

This case highlights that congenital gastrointestinal anomalies should remain on the differential diagnosis in infants with refractory symptoms even in the absence of complete obstruction, or neonatal onset. Early surgical referral, particularly in the presence of radiologic signs of partial obstruction, or poor growth, may prevent unnecessary treatment delays and complications.

## Conclusion

This case illustrates that congenital intestinal anomalies such as type I jejunal atresia with incomplete mucosal diaphragm and concurrent congenital bands can cause subtle, chronic or intermittent presentations beyond the newborn period, with a high risk of misdiagnosis as functional or allergic gastrointestinal syndromes, resulting in delayed surgery and avoidable morbidity. Therefore, structural gut anomalies should be considered in the differential diagnosis of intractable digestive symptoms in babies that do not respond to the therapeutic options for functional and allergic gastrointestinal syndromes, particularly if gastrointestinal X-ray based imaging shows partial small bowel obstruction or poor growth is present.
